# Association of sedentary behaviour and physical activity with cardiometabolic health in Japanese adults

**DOI:** 10.1038/s41598-022-05302-y

**Published:** 2022-02-10

**Authors:** Keita Kinoshita, Naoki Ozato, Tohru Yamaguchi, Motoki Sudo, Yukari Yamashiro, Kenta Mori, Mizuri Ishida, Yoshihisa Katsuragi, Hiroyuki Sasai, Takuji Yasukawa, Koichi Murashita, Shigeyuki Nakaji, Kazushige Ihara

**Affiliations:** 1 Department of Active Life Promotion Sciences, Graduate School of Medicine, Hirosaki University, Aomori, Japan; 2 Health & Wellness Products Research Laboratories, Kao Corporation, Tokyo, Japan; 3 Personal Health Care Products Research Laboratories, Kao Corporation, Tokyo, Japan; 4 Department of Innovation Center for Health Promotion, Graduate School of Medicine, Hirosaki University, Aomori, Japan; 5 Research Team for Promoting Independence and Mental Health, Tokyo Metropolitan Institute of Gerontology, Tokyo, Japan; 6 COI Research Initiatives Organization, Graduate School of Medicine, Hirosaki University, Tokyo, Japan; 7 Department of Social Medicine, Graduate School of Medicine, Hirosaki University, Aomori, Japan

**Keywords:** Disease prevention, Public health, Weight management

## Abstract

Although the Asian population exhibits excessive sedentary behaviour and has a high susceptibility to metabolic syndrome (MetS), the nature of these associations remains unclear. This study aimed to investigate the association of sedentary time with cardiometabolic health and examine the association of reallocating sedentary time to light physical activity (LPA) or moderate-vigorous physical activity (MVPA) on cardiometabolic health in Japanese adults. A cross-sectional study was performed using data obtained from 758 Japanese adults. We assessed sedentary time, LPA, and MVPA using an accelerometer. Linear and logistic regression models were used to analyse the association between sedentary time and cardiometabolic risk factors. An isotemporal substitution model was used to estimate the theoretical influence of reallocating sedentary time to LPA or MVPA. A longer sedentary time was associated with worse cardiometabolic health, including MetS. Reallocating 30 min of sedentary time to LPA was significantly associated with lower body mass index, visceral fat, insulin resistance, triglyceride, and MetS levels and increased muscle mass and HDL-C (all *P* < 0.05). Reallocating 30 min of sedentary time to MVPA was strongly associated with the aforementioned factors. These results demonstrate the potential beneficial effects of reallocating sedentary time to LPA and MVPA on cardiometabolic health of Asians.

## Introduction

Cardiometabolic diseases, including metabolic syndrome (MetS), have been the main cause of mortality in the last two decades worldwide^1^. MetS is a cluster of risk factors such as abdominal obesity, hyperglycaemia, hypertension, and dyslipidaemia, with an increasing worldwide prevalence^2–4^. Globally, there are several MetS definitions^5^, with the criteria for MetS in Japan requiring the presence of abdominal obesity as an essential component^6^. This is partly attributed to Asian individuals having a higher susceptibility to MetS, with visceral fat accumulation even in individuals with lower body mass index (BMI), compared with Western individuals^7,8^.

Cardiometabolic diseases are closely associated with lifestyle-related factors, including dietary habits, sleep, and physical activity. Notably, physical activity has significant health benefits for preventing diseases, whereas a sedentary lifestyle is among the serious, yet insufficiently addressed public health problems. In 2020, the World Health Organization Guidelines on physical activity and sedentary behaviour recommended replacing sedentary time with physical activity, including light physical activity (LPA), for health benefits^9^. In Japan, current public physical activity guidelines are mostly focused on moderate-vigorous physical activity (MVPA)^10^. While there is increasing evidence regarding the association between sedentary time and cardiometabolic health in the Asian population, including Japan^11,12^; the extent of the association of sedentary time with cardiometabolic health, including visceral fat accumulation, and the effect of replacing it with physical activity remains unclear.

Recent epidemiological studies have used the isotemporal substitution model to explore the theoretical influence of reallocating sedentary time to physical activity. This model is among the methods that consider interdependency and has often been used in recent studies on physical activity that have examined various health outcomes, including all-cause mortality^13–15^, depression^16,17^, and cardiometabolic health^18–23^. This approach has facilitated the increasing evidence regarding the benefits of both LPA and MVPA. However, previous findings regarding cardiometabolic health, especially its relationship with LPA, have been inconsistent due to differences among study participants in terms of factors such as race, physical activity levels, and general health condition. Furthermore, although the Asian population exhibits extensive sedentary behaviour^24^, studies using this approach are currently limited to the Western population with none examining the cardiometabolic health in the Asian population. This study aimed to investigate the association of sedentary time with cardiometabolic health including visceral fat, as well as to examine the association of reallocating sedentary time to LPA or MVPA on cardiometabolic health in Japanese adults using an isotemporal substitution analysis.

## Results

In 2018, 1,056 individuals participated in the health check-up. Among them, we excluded 120 participants with incomplete clinical assessments, dietary data, or accelerometer data. Additionally, we excluded 178 participants who did not meet the criteria regarding accelerometer data. Finally, this study included 758 participants (291 men and 467 women) aged 20–88 years.

Table 1 shows the characteristics of participants. Among them, 13.5%, 30.2%, 55.3%, 11.7%, and 24.8% of the participants had MetS, abdominal obesity, hypertension, hyperglycaemia, and dyslipidaemia, respectively. The mean accelerometer wear time was 15.6 h/day. The mean standardised sedentary time was 10.9 h/day, which accounted for 67.9% of the accelerometer wear time. The average time spent on LPA and MVPA was 4.58 and 0.42 h/day, respectively. Supplemental Table 1 presents the participant characteristics grouped according to the quartiles of standardised sedentary time. The higher quartiles showed significantly lower age, LPA, MVPA, and energy intake (All P for trend < 0.001). High alcohol intake was significantly associated with sedentary time (P for trend < 0.001).

**Table 1:** Participant characteristics.

	All (n = 758)
Age (years)	55.0 (14.3)
Female (%)	61.6
Prevalence of cardiometabolic risk factors	
Metabolic syndrome (%)	13.5
Abdominal obesity (%)	30.2
Hypertension (%)	55.3
Hyperglycaemia (%)	11.7
Dyslipidaemia (%)	24.8
Sedentary behaviour and physical activity	
Accelerometer wear time (h/day)	15.6 (1.90)
Sedentary time (h/day)^a^	10.9 (1.37)
LPA(h/day)	4.58 (1.37)
MVPA (h/day)	0.42 (0.24)
Lifestyle/dietary data	
Smoking status (%)	
Never	64.1
Former	22.0
Current	13.9
Alcohol intake (%)	
None	44.9
Low (< 20 g/day)	33.1
High (≥ 20 g/day)	22.0
Energy intake (kcal/day)	1856 (564)

Data shown are means (standard deviations) or percentages.

*LPA* light physical activity, *MVPA* moderate-vigorous physical activity.

Abdominal obesity, visceral fat area ≥ 100 cm^2^; hypertension, systolic blood pressure ≥ 130 mmHg, and/or diastolic blood pressure ≥ 85 mmHg; hyperglycaemia, fasting brood glucose ≥ 110 mg/dL; and dyslipidaemia, triglyceride ≥ 150 mg/dL and/or high-density lipoprotein cholesterol < 40 mg/dL.

^a^Sedentary time was expressed as the estimated hours of sedentary time per day given as standardised 16 h of accelerometer wear time.

Table 2 shows the association between the quartiles of sedentary time and cardiometabolic risk factors based on multiple linear regression analysis. Higher sedentary time was significantly associated with higher visceral fat area (VFA) levels, HOMA-IR, and TG levels as well as lower levels of total body muscle mass and HDL-C (all P for trend < 0.001). There were no significant associations of sedentary time with BMI, systolic blood pressure (SBP), diastolic blood pressure (DBP), blood glucose, and LDL-C.

**Table 2:** Associations of sedentary time with cardiometabolic risk components.

	Q1 (n = 189) < 10.0 h	Q2 (n = 190) 10.0–10.9 h	Q3 (n = 190) 10.9–11.8 h	Q4 (n = 189) ≥ 11.8 h	*P* for trend
BMI (kg/m^2^)	22.7 (22.2;23.2)	22.8 (22.3;23.2)	22.7 (22.2;23.2)	23.4 (22.9;23.9)	0.081
VFA (cm^2^)	76.5 (70.9;82.0)	77.8 (72.4;83.1)	81.8 (76.4;87.1)	93.5 (87.9; 99.0)	< 0.001
Muscle mass (%)	70.9 (69.6;71.4)	70.5 (69.6;71.4)	69.7 (68.8;70.6)	67.8 (66.9;68.7)	< 0.001
SBP (mmHg)	127 (125;130)	125 (123;128)	124 (122;127)	127 (124;129)	0.783
DBP (mmHg)	80.3 (78.6;82.20)	78.6 (77.0;80.3)	77.9 (76.2;79.5)	79.3 (77.6;80.9)	0.326
Glucose^a^ (mg/dL)	95.2 (93.5;97.0)	93.1 (91.6;94.8)	92.0 (90.5;93.6)	96.0 (94.2;97.6)	0.821
HOMA-IR^a^ (mg/dL)	1.10 (1.02;1.19)	1.05 (0.97;1.13)	1.10 (1.02;1.18)	1.34 (1.24;1.45)	< 0.001
TG^a^ (mg/dL)	77.9 (72.4;83.9)	77.1 (71.8;82.7)	84.5 (78.8;90.7)	93.1 (86.6;100)	< 0.001
HDL-C (mg/dL)	70.0 (67.6;72.4)	67.5 (65.2;69.8)	66.8 (64.6;69.1)	61.7 (59.3;64.0)	< 0.001
LDL-C (mg/dL)	120 (116;124)	118 (114;122)	119 (114;123)	120 (116;125)	0.796

Values shown are adjusted means (95% confidence intervals).

Linear regression models were used, with adjustments for age, sex, smoking status, alcohol intake, energy intake, and moderate-vigorous physical activity.

*BMI* body mass index, *VFA* visceral fat area, *SBP* systolic blood pressure, *DBP* diastolic blood pressure, *HOMA-IR* homeostasis model assessment of insulin resistance, *TG* triglyceride, *HDL-C* high-density lipoprotein cholesterol, *LDL-C* low-density lipoprotein cholesterol.

^a^Log-transformed values were used, and the adjusted means were back-transformed.

Table 3 shows the aOR (95% CI) for MetS and its components grouped according to the quartiles of sedentary time. There were significant associations of sedentary time with MetS (P for trend = 0.004), abdominal obesity (P for trend < 0.001), and dyslipidaemia (P for trend = 0.018). Compared with the lowest quartile (< 10.0 h/day), the highest quartile of sedentary time (≥ 11.8 h/day) showed a significantly higher aOR of MetS (aOR = 2.52 [95% CI 1.34, 4.73]), abdominal obesity (aOR = 2.79 [95% CI 1.63, 4.75]), and dyslipidaemia (aOR = 1.81 [95% CI 1.08, 3.04]). There were no significant associations between MetS and its components in the second (10.0–10.9 h/day) and third quartiles (10.9–11.8 h/day) compared with the lowest quartile.

**Table 3:** Associations of sedentary time with metabolic syndrome and its components.

	Q1 (n = 189) < 10.0 h	Q2 (n = 190) 10.0–10.9 h	Q3 (n = 190) 10.9–11.8 h	Q4 (n = 189) ≥ 11.8 h	*P* for trend
Metabolic syndrome	1.00 (reference)	0.73 (0.36;1.47)	0.98 (0.51;1.88)	2.52 (1.34;4.73)	0.004
Abdominal obesity	1.00 (reference)	1.02 (0.60;1.74)	1.29 (0.77;2.16)	2.79 (1.63; 4.75)	< 0.001
Hypertension	1.00 (reference)	1.21 (0.75;1.96)	0.97 (0.60;1.58)	1.31 (0.78;2.19)	0.510
Hyperglycaemia	1.00 (reference)	0.74 (0.38;1.44)	0.56 (0.27;1.13)	1.72 (0.90;3,28)	0.219
Dyslipidaemia	1.00 (reference)	1.16 (0.70;1.92)	1.42 (0.86;2.33)	1.81 (1.08;3.04)	0.018

Values shown are adjusted odds ratios (95% confidence intervals). Logistic regression models were used, with adjustment for age, sex, smoking status, alcohol intake, energy intake, and moderate-vigorous physical activity.

Abdominal obesity: visceral fat area ≥ 100  cm^2^; hypertension: systolic blood pressure ≥ 130 mmHg, and/or diastolic blood pressure ≥ 85 mmHg or using antihypertensive drugs; hyperglycaemia: fasting brood glucose ≥ 110 mg/dL or using antidiabetic drugs; and dyslipidaemia: triglycerides ≥ 150 mg/dL and/or high-density lipoprotein cholesterol < 40 mg/dL or using antihyperlipidemic drugs.

**Figure 1:**
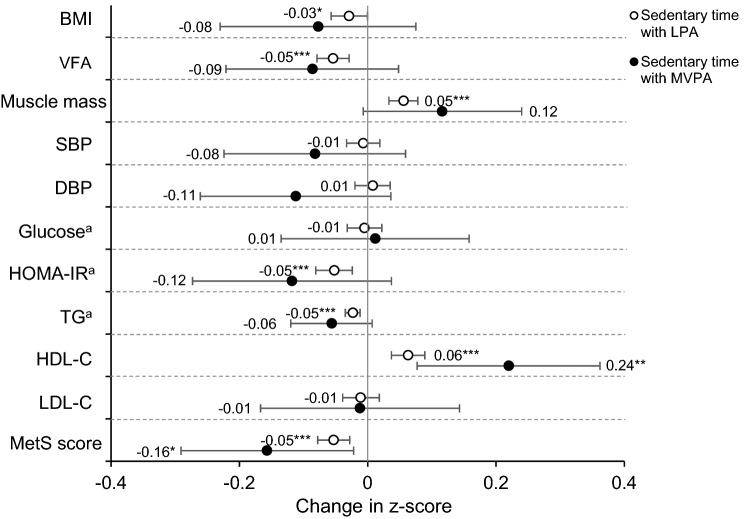
Isotemporal substitution of sedentary time with LPA or MVPA on cardiometabolic risk factors. The values shown are β (95% confidence interval). Each z-score indicates the amount of change in the outcome variable associated with reallocating 30 min of sedentary time to LPA (open circle) or MVPA (closed circle). Abbreviations: BMI, body mass index; VFA, visceral fat area; SBP, systolic blood pressure; DBP, diastolic blood pressure; HOMA-IR, homeostasis model assessment of insulin resistance; TG, triglycerides; HDL-C, high-density lipoprotein cholesterol; LDL-C, low-density lipoprotein cholesterol. Linear regressions models were used to assess isotemporal substitution of sedentary time with LPA or MVPA, with adjustment for age, sex, smoking status, alcohol intake, energy intake, and accelerometer wear time. ^a^Log-transformed value was used. The MetS score was calculated by standardising and summing VFA, blood pressure ([SBP + DBP]/2), log glucose, log TG, and inverse HDL-C. *P < 0.05, **P < 0.01, ***P < 0.001.

Figure 1 and Supplemental Table 2 show the results of the isotemporal substitution models for cardiometabolic risk factors. In Fig. 1, reallocating 30 min of sedentary time to LPA was significantly associated with lower BMI, VFA, HOMA-IR, TG, and MetS scores, as well as a higher total body muscle mass and HDL-C (all p-value < 0.05). Similar results were yielded in the single-factor model for LPA (Supplemental Table 3). Reallocating 30 min of sedentary time to MVPA, rather than LPA, showed stronger associations with these cardiometabolic risk factors. Contrastingly, the single-factor model for MVPA showed significant associations with VFA, total body muscle mass, HOMA-IR, TG, HDL-C, and MetS score (Supplemental Table 3).

## Discussion

This study on Japanese adults reported a significant association of longer sedentary time with worse cardiometabolic health. Moreover, reallocating sedentary time to LPA was consistently associated with better cardiometabolic health. Previous studies using the isotemporal substitution model to examine the effect of reallocating sedentary time to other physical activity on health outcomes have primarily focused on Westerners^25^. However, previous findings regarding cardiometabolic health, especially its relationship with LPA, have been inconsistent due to differences among study participants^18–23^. Therefore, there is a need for studies on target populations to facilitate more specific public health recommendations for preventing cardiometabolic diseases. This study contributes toward evidence for reducing sedentary behaviour and encouraging physical activity, including LPA, in the Asian population using the isotemporal substitution model.

We observed a significant association of objectively measured sedentary time with visceral fat accumulation in the Japanese population. This is consistent with previous findings regarding objectively measured sedentary time and visceral fat per se in the Western population^22,26–28^, even though the Asian population spends more time in sedentary behaviour^24^ and has a higher susceptibility to visceral fat accumulation^7,8^ compared with the Western population. Regarding BMI, several studies have reported significant associations with sedentary time^12^; however, we did not observe a significant association. BMI is widely used as a general obesity indicator; however, although it reflects both fat content and muscle mass^29^, it cannot provide a separate measurement for both. There was an inverse association of sedentary time with total body muscle mass, which partly explains our results regarding BMI. Taken together, reducing sedentary time might be one of the common approaches to address increasing abdominal obesity worldwide.

Our results are consistent with those of previous reports of an association of longer sedentary time with worse cardiometabolic health, including dyslipidaemia, insulin resistance, and MetS besides visceral fat^11,22,30^. This could be attributed to the potential mechanisms underlying the sedentarism effect^31^. Specifically, prolonged sedentary behaviour may reduce muscle contraction and promote ectopic fat storage, which causes insulin resistance and dyslipidaemia. Recently, prolonged sedentary time has been associated with a greater risk of cardiovascular disease and all-cause mortality^32,33^. These findings confirm that increased sedentary behaviour is associated with worse health outcomes, including cardiometabolic health.

We found that reallocating sedentary time to LPA had beneficial associations on MetS score, BMI, VFA, TG levels, HDL-C levels, and HOMA-IR. A systematic review by Amagasa et al. reported that objectively measured LPA was associated with several cardiometabolic risk factors, including waist circumference, TG levels, insulin levels, and MetS occurrence^34^. Additionally, they also reported an inconsistent association of LPA with BMI and HDL-C, as well as insufficient evidence regarding VFA and HOMA-IR. Our single-factor model findings contribute to existing knowledge by adding the importance of increasing LPA on these risk factors. Moreover, recent epidemiological studies have used the isotemporal substitution model, which facilitates the interpretation of the interrelationships between different movement-related behaviours and their relationships with health compared with typical analytic models, including single-factor models. A more recent longitudinal study conducted by Whitaker et al. reported an association of reallocating sedentary time to LPA or MVPA with improved cardiometabolic health after 10 years in black and white men and women^21^. Specifically, there was a significant association of reallocating sedentary time to LPA with a lower composite cardiometabolic risk score, which was primarily characterised by a lower waist circumference and insulin levels, as well as higher HDL-C levels, which is consistent with our findings. LPA accounts for a considerable proportion of the total daily activity; moreover, it has been estimated that reallocating 1 h of sedentary time to LPA increases energy expenditure by approximately 3%, which could have significant health benefits^14,18^. However, compared with MVPA, there have been fewer longitudinal and intervention studies on LPA. Therefore, there is a need for further studies to confirm the dose-dependent effects of reallocating sedentary time to LPA in several races/ethnicities, including the Asian population.

In this study, we confirmed the effects of MVPA on cardiometabolic health in single-factor models, which is consistent with a previous study^10^. Moreover, we examined the impact of reallocating sedentary time to MVPA on cardiometabolic health. Compared with LPA, reallocating to MVPA had a greater effect on the MetS score; however, there were fewer cardiometabolic risk components significantly associated with MVPA, which is consistent with the findings by Whitaker et al.^21^. This can be partly attributed to the MVPA duration in the study population. The MVPA durations in the present and Whitaker’s study were approximately 25 min/day and 29.8 min/day, which are close to each other. Additionally, Healy et al. reported non-significant associations of MVPA with cardiometabolic biomarkers for overweight or obese adults with type 2 diabetes, whose MVPA was 17.9 min/day^20^. Although between-study comparisons of physical activity levels are impeded by methodological differences in accelerometer measurement, the theoretical effect of reallocating sedentary time to MVPA might be dependent on the original MVPA levels in study participants. These results suggest that specific guidelines adjusted for the physical activity levels of target populations might be needed.

The strengths of this study include the relatively large sample size with a wide age range, obtaining VFA measurements using an abdominal bio-impedance method, and objective measurement of sedentary time and physical activity using an accelerometer. Additionally, activity was measured for > 7 days (≥ 10 h/day) with 4-s epochs, which allowed the reflection of actual daily activity^35^. Our results could be applicable for the improvement of cardiometabolic health in sedentary populations, such as the Asian population; this could be achieved by monitoring daily activity and encouraging reallocating sedentary time not only to MVPA but also to LPA. However, this study also had some limitations. First, given its cross-sectional design, this study could not establish a strong causal relationship. Isotemporal substitution does not reflect real-time reallocation. Furthermore, the influence of age on the association between sedentary behaviour and morbidities should be considered in the amount of reallocation of sedentary time. Moreover, there is a need for longitudinal studies or intervention trials to determine the causality, amount of reallocation time, and underlying mechanisms. Second, the loss of participants due to insufficient accelerometer data could have led to selection bias. Participants who did not adhere to wearing the accelerometer were more likely to be male and younger; however, cardiometabolic health was comparable among participants. Third, there might have been an overestimation of the sedentary time since sedentary behaviour was defined based on levels (≤ 1.5 METs), which cannot distinguish between sitting and standing postures by this study device. Although the term “stationary” refers to waking behaviours such as lying, reclining, sitting, or standing^36^, previous accelerometer-based studies with a similar measurement method as ours (based on levels [≤ 1.5 METs]) have used the term “sedentary”^30,32,37,38^. Therefore, we used the term “sedentary” to ensure comparability. Finally, although we adjusted for several covariates, there might have been residual confounding variables, including sleep. Wang et al. reported that short sleep duration was associated with an increased risk of cardiovascular heart disease^39^. Therefore, future studies should consider 24-h activity measurement.

In conclusion, there was a significant association of longer sedentary time with worse cardiometabolic health; moreover, reallocating 30 min of sedentary time to LPA was consistently associated with better cardiometabolic health in Japanese adults. This study adds to the current research body using the isotemporal substitution model, which has been scarcely applied in the Asian population. Additionally, these results highlight the potential beneficial effects of encouraging both LPA and MVPA on cardiometabolic health. Further longitudinal or intervention studies are required to confirm the effectiveness of reallocating sedentary time to LPA on cardiometabolic health.

## Methods

### Participants

The Iwaki Health Promotion Project was launched in 2005. Moreover, an annual health check-up has been conducted as part of the activities of the project for adults living in the Iwaki region of the Hirosaki City in the Aomori Prefecture, Japan^40–44^. All adult residents (≈ 10,000) in this region were invited on the basis of the resident registration, with approximately 10% of these adult residents voluntarily participating in the health check-up annually. This population-based cross-sectional study analysed data obtained from the health check-up conducted between May 27 and June 5, 2018, on 1,056 individuals. This study was approved by the Ethics Committee of Hirosaki University School of Medicine (2018–012, 2018–063) and conducted in accordance with the principles of the Declaration of Helsinki. All the study participants provided written informed consent. This study was registered in the University Hospital Medical Information Network (UMIN-CTR, https://www.umin.ac.jp, UMIN ID: UMIN000036741).

### Measurement of sedentary time and physical activity

Sedentary time and physical activity were measured using an accelerometer (HW-100, Kao Corporation, Tokyo, Japan), which allows 40 days of continuous recording at a sampling frequency of 64 Hz. The epoch length of the accelerometer was 4 s. The activity intensity level was measured as previously described^45–47^. Briefly, accelerometer data were calculated as the time spent in each of the following three intensity levels: 1) sedentary behaviour, ≤ 1.5 metabolic equivalent tasks (METs); 2) LPA, 1.6–2.9 METs; and 3) MVPA, ≥ 3 METs. A period of ≥ 35 min where activity was not recorded using an accelerometer was designated as non-wear time. Sedentary time, LPA, and MVPA were expressed as the mean daily hours across all adherent days (wearing ≥ 10 h/day) for all participants.

The participants were instructed to wear the HW-100 on the waists throughout their awake period, except during swimming or bathing, as well as to maintain their usual activities. Additionally, the participants were instructed to begin wearing the HW-100 promptly after completing their health check-up and to return it after 10 days. The criterion for analysis was wearing the accelerometer for a total duration of ≥ 7 days (≥ 10 h per day) during the first 10 days after starting to wear the accelerometer.

### Cardiometabolic risk factors

VFA was measured using a bioimpedance-type visceral fat meter (EW-FA90; Panasonic Corporation, Osaka, Japan), which is a certified medical device in Japan (No. 22500BZX00522000) for non-invasive VFA measurement^48^. Measurements obtained using this device are strongly correlated with those obtained using computed tomography^49^, which is the gold standard for VFA measurement. Total body muscle mass (expressed as a percentage of the total body mass) was measured using Impedance Analyzer (MC180, Tanita Corporation, Tokyo, Japan)^50^.

Morning blood samples were collected from the peripheral veins after ≥ 9 h fasting state. Blood glucose, insulin, triglyceride (TG), high-density lipoprotein cholesterol (HDL-C), and low-density lipoprotein cholesterol measurements were performed by LSI Medience Co. (Tokyo, Japan) according to their standard operating procedure. Homeostatic model assessment of insulin resistance (HOMA-IR) was calculated using the following equation: fasting blood glucose × fasting insulin/405.

### Metabolic syndrome

The Japanese criteria for MetS were used to determine the MetS prevalence^6,51^. These criteria included abdominal obesity, which was defined as VFA ≥ 100 cm^2^, as an essential component, plus more than two of the following factors: hypertension, which was defined as SBP ≥ 130 mmHg, DBP ≥ 85 mmHg, or using antihypertensive drugs; hyperglycaemia, which was defined as fasting blood glucose ≥ 110 mg/dl or using antidiabetic drugs; and dyslipidaemia, which was defined as TG ≥ 150 mg/dl, HDL-C < 40 mg/dl, or using antihyperlipidemic drugs. The MetS score, which was represented as a continuous variable, was estimated by standardising and summing the following MetS components: VFA, blood pressure [(zSBP + zDBP)/2], fasting blood glucose, TG, and inverted zHDL-C, as previously described^11^.

### Medication, smoking, and alcohol consumption

We obtained data regarding medication (hypertension, diabetes, and dyslipidaemia) and smoking habits using self-administered questionnaires prepared for the health check-up. Daily alcohol intake and total energy intake were determined using the Brief Diet History Questionnaire^52,53^. Alcohol intake was categorised as none, low intake (< 20 g/day), and high intake (≥ 20 g/day).

### Statistical analysis

Given the strong correlation between accelerometer wear time and sedentary time (r = 0.72) and the differences in the accelerometer wear time, we standardised the sedentary time to 16 h per day of accelerometer wear time using residuals obtained when regressing sedentary time on accelerometer wear time as previously described^30,32,37^. The participants were categorised into quartiles based on the standardised sedentary time as follows: < 10.0 h, 10.0 h ≤ sedentary time < 10.9 h, 10.9 h ≤ sedentary time < 11.8 h, and sedentary time ≥ 11.8 h.

The participant characteristics are reported as means ± standard deviation or percentage. Continuous and categorical variables were compared among the quartile groups using the Jonckheere‒Terpstra trend test and Cochran‒Armitage trend test, respectively.

Multiple linear regression analysis was used to assess the association of sedentary time with cardiometabolic risk factors, which was reported as adjusted means (95% confidence interval [CI]) for each quartile of sedentary time. Due to their skewed distribution, blood glucose, HOMA-IR, and TG levels were log-transformed, followed by back-transformation of the adjusted means to yield adjusted geometric means. Multiple logistic regression analysis was used to determine the adjusted odds ratio (aOR) and 95% CI for MetS and its components, which compared the upper and lower quartile groups. These regression analyses were adjusted for sex, age, alcohol intake, smoking status, total energy intake (kcal/d), and MVPA. We assessed linear trends across quartiles by including each participant’s quartile as an ordinal variable in the regression analysis. All regression models were checked for linearity, normality, and homoscedasticity. Variance inflation factors for multicollinearity in the regression analysis were confirmed to be < 5.

We used an isotemporal substitution model to quantify the associations of reallocating 10 or 30 min of sedentary time to LPA or MVPA with cardiometabolic risk factors. For the isotemporal substitution models, the accelerometer wear time was entered simultaneously with LPA and MVPA, with subsequent adjustment for the aforementioned covariates^54^. The outcome variables were standardised as z-scores for better elucidation of the reallocation effect. The resulting regression coefficient represented the association of reallocating sedentary time to LPA and MVPA. For a better interpretation of the isotemporal analysis results, we also performed linear regression models without adjustment for other activity categories (i.e., single-factor models).

Statistical tests were two-tailed; moreover, statistical significance was set at *P* < 0.05 or *P* < 0.01 for interactions (considering multiple hypothesis testing). As interactions by age and sex with sedentary behaviour were not statistically significant, pooled analyses were conducted. All analyses were performed using SPSS (version 25; SPSS Inc., Chicago, IL, USA) and the R environment (version 3.6.2; R Core Team, Vienna, Austria).

## Supplementary Information

Supplementary Information.
