# Supplementary material for: Association of sedentary behaviour and physical activity with cardiometabolic health in Japanese adults
Source: Sci Rep. 2022 Feb 10;12:2262. doi: 10.1038/s41598-022-05302-y (PMC8831565; doi:10.1038/s41598-022-05302-y)
Supplement: Supplementary file 1 — Supplementary Information. [file 41598_2022_5302_MOESM1_ESM.docx]

Supplemental Table 1. Characteristics of the participants across the quartiles of sedentary time

|  | Q1 (n = 189) < 10.0 h | Q2 (n = 190) 10.0-10.9 h | Q3 (n = 190) 10.9-11.8h | Q4 (n = 189) ≥11.8h | *P* for trend |
| --- | --- | --- | --- | --- | --- |
| Age (year) | 57.4 (11.2) | 55.7 (13.0) | 55.7 (14.9) | 51.2 (16.8) | <0.001 |
| Woman (%) | 50.8 | 71.1 | 60.5 | 64.0 | 0.066 |
| Sedentary behavior and physical activity | | | | | |
| Accelerometer wear time (h/day) | 15.6 (1.70) | 15.5 (1.99) | 15.5 (1.95) | 15.6 (1.98) | 0.510 |
| Sedentary time (h/day)^a^ | 9.04 (0.83) | 10.5 (0.26) | 11.4 (0.26) | 12.5 (0.50) | <0.001 |
| LPA(h/day) | 6.28 (0.91) | 4.89 (0.56) | 4.09 (0.55) | 3.04 (0.65) | <0.001 |
| MVPA (h/day) | 0.54 (0.25) | 0.44 (0.24) | 0.38 (0.22) | 0.33 (0.19) | <0.001 |
| Lifestyle/dietary data |  |  |  |  |  |
| Smoking status (%) |  |  |  |  |  |
| Never | 15.0 | 17.0 | 16.0 | 16.1 | 0.588 |
| Former | 5.94 | 5.41 | 5.80 | 4.88 | 0.410 |
| Current | 3.96 | 2.64 | 3.30 | 3.96 | 0.814 |
| Alcohol intake (%) |  |  |  |  |  |
| None | 9.89 | 11.7 | 11.1 | 12.1 | 0.133 |
| Low (<20 g/day) | 7.65 | 7.78 | 8.58 | 9.10 | 0.178 |
| High (>20 g/day) | 7.39 | 5.54 | 5.41 | 3.69 | <0.001 |
| Energy intake (kcal/day) | 1993 (568) | 1828 (586) | 1875 (554) | 1727 (517) | <0.001 |

Data are presented as mean (standard deviation) or percentages.

Abbreviation: *LPA*, light physical activity; *MVPA*, moderate-vigorous physical activity.

Jonkeere-Terpstra trend test or Cochran-Armitage trend test was used. ^a^Sedentary time was expressed as the estimated hours of sedentary time per day within a standardized 16 h of accelerometer wear time.

Supplemental Table 2. Isotemporal substitution of sedentary time with LPA or MPVA on cardiometabolic risk components

|  | Sedentary time with LPA | | *P* | Sedentary time with MVPA | | *P* |
| --- | --- | --- | --- | --- | --- | --- |
|  | Per 10 minutes | Per 30 minutes |  | Per 10 minutes | Per 30 minutes |  |
| BMI | -0.03 (-0.06;-0.00) | -0.10 (-0.19;-0.00) | 0.047 | -0.09 (-0.26;0.09) | -0.26 (-0.78;0.26) | 0.321 |
| VFA | -0.79 (-1.16;-0.43) | -2.38 (-3.47;-1.29) | <0.001 | -1.26 (-3.23;0.71) | -3.79 (-9.70;2.12) | 0.208 |
| Muscle mass | 0.14 (0.08;0.20) | 0.43 (0.25;0.61) | <0.001 | 0.30 (-0.02;0.62) | 0.90 (-0.06;1.86) | 0.066 |
| SBP | -0.04 (-0.20;0.12) | -0.13 (-0.61;0.36) | 0.612 | -0.51 (-1.39;0.37) | -1.53 (-4.17;1.10) | 0.255 |
| DBP | 0.03 (-0.08;0.14) | 0.09 (-0.24;0.43) | 0.576 | -0.45 (-1.06;0.15) | -1.36 (-3.17;0.44) | 0.138 |
| Glucose^a^ | 1.00 (1.00;1.00) | 1.00 (1.00;1.00) | 0.706 | 1.00 (0.99;1.01) | 1.00 (0.98;1.02) | 0.876 |
| HOMA-IR^a^ | 0.99 (0.99;1.00) | 0.97 (0.96;0.99) | <0.001 | 0.98 (0.95;1.01) | 0.94 (0.86;1.02) | 0.137 |
| TG^a^ | 0.99 (0.99;1.00) | 0.97 (0.96;0.99) | <0.001 | 0.98 (0.95;1.00) | 0.93 (0.86;1.01) | 0.079 |
| HDL-C | 0.37 (0.22;0.52) | 1.11 (0.65;1.57) | <0.001 | 1.29 (0.45;2.12) | 3.86 (1.36;6.35) | 0.003 |
| LDL-C | -0.10 (-0.39;0.18) | -0.31 (-1.16;0.53) | 0.465 | -0.12 (-1.64;1.40) | -0.35 (-4.91;4.21) | 0.880 |
| MetS score | -0.06 (-0.09;-0.03) | -0.18 (-0.26;-0.09) | <0.001 | -0.17 (-0.32;-0.02) | -0.52 (-0.97;-0.07) | 0.023 |

Values shown are β or relative rates (95% confidence interval). Each estimate indicates the amount of change in the outcome variable associated with reallocating 10 or 30 min of sedentary time to LPA and MVPA.

Abbreviations: BMI, body mass index; VFA, visceral fat area; SBP, systolic blood pressure; DBP, diastolic blood pressure; HOMA-IR, homeostasis model assessment of insulin resistance; TG, triglyceride; HDL-C, high density lipoprotein cholesterol; LDL-C, low density lipoprotein cholesterol; MetS, metabolic syndrome; LPA, light physical activity; MVPA, moderate-vigorous physical activity.

Linear regressions models were used with adjustment for age, sex, smoking status, alcohol intake, energy intake, and accelerometer wear time.

^a^Log-transformed values were back transformed as relative rates. Relative rates > 1 and < 1 indicate an increase and decrease, respectively, in mean cardiometabolic risk components. The MetS score was calculated by standardizing and summing VFA, blood pressure ([SBP+DBP]/2), log glucose, log insulin, log triglycerides, and inverse HDL-C.

Supplemental Table 3. Association of LPA or MVPA with cardiometabolic risk components

|  | LPA | *P* | MVPA | *P* |
| --- | --- | --- | --- | --- |
| BMI | -0.11 (-0.20;-0.01) | 0.023 | -0.38 (-0.89;0.12) | 0.137 |
| VFA | -2.54 (-3.60;-1.48) | <0.001 | -6.76 (-12.6;-0.94) | 0.023 |
| Muscle mass | 0.47 (0.30;0.64) | <0.001 | 1.44 (0.49;2.39) | 0.003 |
| SBP | -0.19 (-0.66;0.28) | 0.429 | -1.69 (-4.25;0.88) | 0.197 |
| DBP | 0.04 (-0.29;0.36) | 0.823 | -1.25 (-3.00;0.51) | 0.164 |
| Glucose^a^ | 1.00 (1.00;1.00) | 0.726 | 1.00 (0.98;1.02) | 0.944 |
| HOMA-IR^a^ | 0.97 (0.95;0.98) | <0.001 | 0.91 (0.83;0.98) | 0.019 |
| TG^a^ | 0.97 (0.96;0.98) | <0.001 | 0.90 (0.83;0.97) | 0.007 |
| HDL-C | 1.27 (0.82;1.72) | <0.001 | 5.24 (2.77;7.70) | <0.001 |
| LDL-C | -0.33 (-1.15;0.49) | 0.431 | -0.74(-5.18;3.70) | 0.742 |
| MetS score | -0.20 (-0.28;-0.12) | <0.001 | -0.74(-1.18;-0.30) | 0.001 |

Values shown are β or relative rates (95% confidence interval). LPA and MVPA expressed as 30 min units. Abbreviations: BMI, body mass index; VFA, visceral fat area; SBP, systolic blood pressure; DBP, diastolic blood pressure; HOMA-IR, homeostasis model assessment of insulin resistance; TG, triglyceride; HDL-C, high density lipoprotein cholesterol; LDL-C, low density lipoprotein cholesterol; MetS, metabolic syndrome; LPA, light physical activity; MVPA, moderate-vigorous physical activity.

Linear regressions models were used with adjustment for age, sex, smoking status, alcohol intake, energy intake, and accelerometer wear time. ^a^Log-transformed values were back transformed as relative rates. Relative rate > 1 and < 1 indicates an increase and decrease, respectively, in mean cardiometabolic risk components. MetS score was calculated by standardizing and summing the VFA, blood pressure ([SBP+DBP]/2), log glucose, log insulin, log triglycerides, and inverse HDL-C.
